# Classification of Corrosion Severity in SPCC Steels Using Eddy Current Testing and Supervised Machine Learning Models

**DOI:** 10.3390/s24072259

**Published:** 2024-04-02

**Authors:** Lian Xie, Prashanth Baskaran, Artur L. Ribeiro, Francisco C. Alegria, Helena G. Ramos

**Affiliations:** Instituto de Telecomunicações, Instituto Superior Técnico, Universidade de Lisboa, 1049-001 Lisbon, Portugal; lian.xie@tecnico.ulisboa.pt (L.X.); prashanth.baskaran@tecnico.ulisboa.pt (P.B.); arturlr@tecnico.ulisboa.pt (A.L.R.); francisco.alegria@tecnico.ulisboa.pt (F.C.A.)

**Keywords:** classification, corrosion, generative and discriminative models, traditional eddy current testing

## Abstract

Steel Plate Cold-Rolled Commercial (SPCC) steel is known to have long-term durability. However, it still undergoes corrosion when exposed to corrosive environments. This paper proposes an evaluation method for assessing the corrosion level of SPCC steel samples using eddy current testing (ECT), along with two different machine learning approaches. The objective is to classify the corrosion of the samples into two states: a less corroded state (state-1) and a highly corroded state (state-2). Generative and discriminative models were implemented for classification. The generative classifier was based on the Gaussian mixture model (GMM), while the discriminative model was based on the logistic regression model. The features used in the classification models are the peaks of the perturbated magnetic fields at two different frequencies. The performance of the classifiers was evaluated using metrics such as absolute error, accuracy, precision, recall, and F1 score. The results indicate that the GMM model is more conducive to categorizing states with higher levels of corrosion, while the logistic regression model is helpful in estimating states with lower levels of corrosion. Meanwhile, high classification accuracy can be achieved based on both methods using eddy current testing.

## 1. Introduction

Steel Plate Cold-Rolled Commercial (SPCC) denotes a steel variant that has undergone a cold-rolling process, enhancing its strength and hardness. Predominantly, it is utilized in sectors demanding precise shapes and dimensions, such as automotive manufacturing [1]. Notably, SPCC offers better corrosion resistance compared to hot-rolled steels, making it a preferred choice for applications exposed to harsh environmental conditions. However, when a material corrodes, it undergoes oxidation and other chemical changes that lead to the loss of material, formation of surface irregularities, and degradation of its mechanical properties [2,3]. These changes can include the loss of structural integrity, reduced tensile strength, and increased susceptibility to fractures.

Over time, the cumulative effects of corrosion can significantly weaken the material, potentially leading to structural failure or diminished performance in various applications. The economic loss due to corrosion is up to 5% of Gross Domestic Product (GDP) in several countries including Japan, the United Kingdom, and the United States [4]. Hence, the detection and classification of corrosion severity play a vital role in ensuring the safety, reliability, and performance of assets and infrastructures. Many researchers have demonstrated the formation and impact of corrosion on mechanical properties under different conditions [5,6,7], and classified corrosion into different states according to their severity.

Nondestructive testing techniques such as radiography, acoustic emission, and thermography have been used for the monitoring of the material’s properties and the characterization of corrosion. Acoustic emission technique has been used by Fregonese et al. [8] to study the development of pitting corrosion on AISI 316L austenitic stainless steel. They found that during the initiation of the pit, the emission was not very significant. However, as the pits propagated, the activity of emission increased. Edalti et al. [9] used tangential radiography (TRT) and double walled radiography (DWT) to obtain the thickness profiles of various steel pipes that have undergone corrosion. The other means of detection and monitoring of corrosion is based on electromagnetic methods.

Electromagnetic methods like eddy current testing have also been used to detect corrosion [10,11]. Frankowski et al. validated the feasibility of eddy current testing for the detection of both uniform and localized corrosion in reinforcing bars [12], while Yusa et al. [13] successfully applied eddy current testing for the detection of fatigue cracks and pressure corrosion cracks. Li et al. [14] used pulsed modulation eddy current testing to detect subsurface corrosion. In addition, the eddy current pulsed thermography technique is also used to detect corrosion in paint coatings [15,16,17].

Investigations pertaining to the detection and monitoring of corrosion have paved the way for their characterization. Characterization of the corroded regions is necessary because small pits on the surface of the material pose little threat to the materials. On the other hand, the presence of deeper pits forces the replacement of the material. One method to characterize the extent of corrosion under coatings was performed by Kopf et al. [18]. It was based on binarizing the thermal images obtained from the pulsed thermography technique, yielding an absolute error of less than 3% in quantifying the extent of corrosion. Alamin et al. [19] employed pulsed eddy current testing for corrosion detection in mild steel, and based on principal component analysis, classified and characterized the corrosion. Postolache et al. [20] utilized a giant magnetoresistor (GMR) to detect changes in the magnetic field for defect characterization, followed by defect classification using neural networks. Nevertheless, the exploration of classifying corrosion through eddy current testing remains ongoing. As the severity of corrosion progresses with time, more data are required for a regression model to estimate the corrosive state of a structure. Therefore, instead of estimating the quantitative dimension of corrosion, it is also common to classify the severity of corrosion into several categories, such as poor and fair [21,22,23]. Hence this paper aims to detect corrosion using eddy current testing and classify the corrosive state into a less corroded state (state-1) and a highly corroded state (state-2), using a generative model and a discriminative model.

The content of this paper is organized as follows. In Section 2, the experimental setup and the experiments carried out to detect the corrosion based on eddy current testing are detailed, with emphasis on measuring the magnetic field components at two different frequencies, and the extraction of features for the subsequent classification models are presented. Section 3 describes the supervised generative and discriminative classifiers. The test samples were classified into two states, and their class-wise performances were evaluated using metrics such as accuracy, precision, recall, and F1 scores. In Section 4, some important conclusions about the classification models are discussed.

## 2. Experiments and Metrology

### 2.1. SPCC Steel Samples

**Figure 1:**
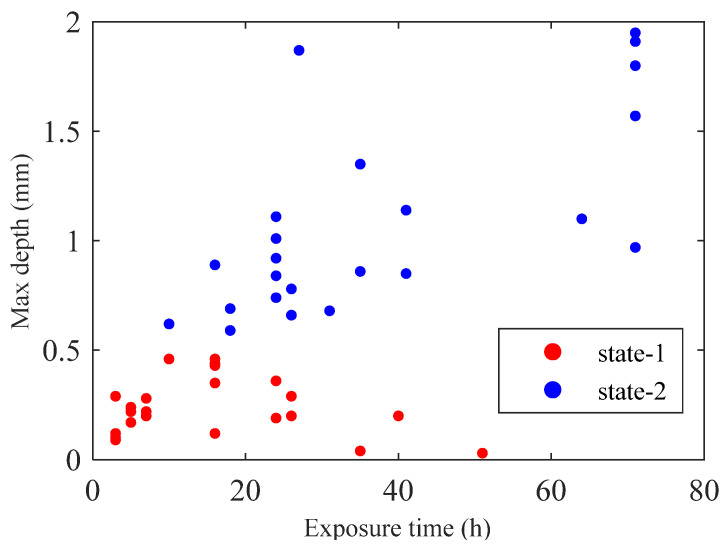
Exposure time and the maximum depth in the corroded area of samples.

**Figure 2:**
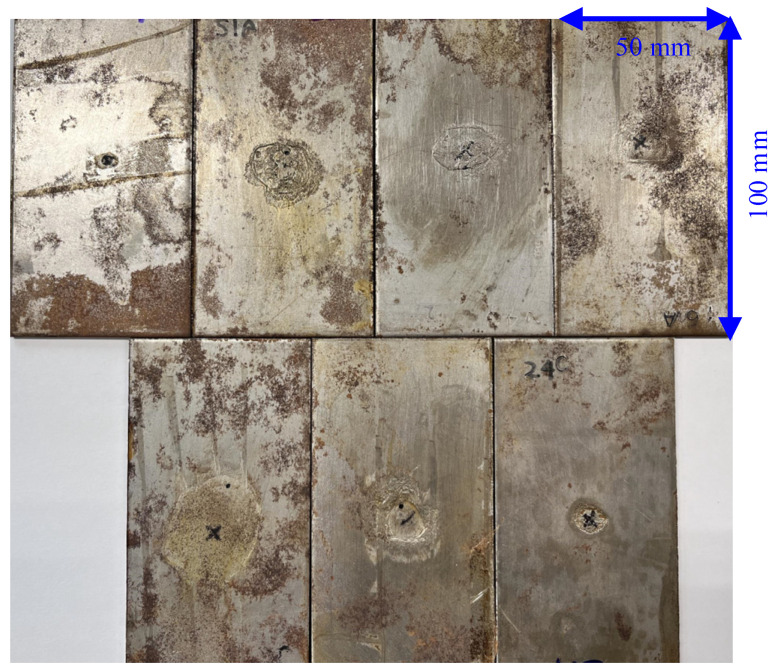
Samples with less corroded state (state-1).

**Figure 3:**
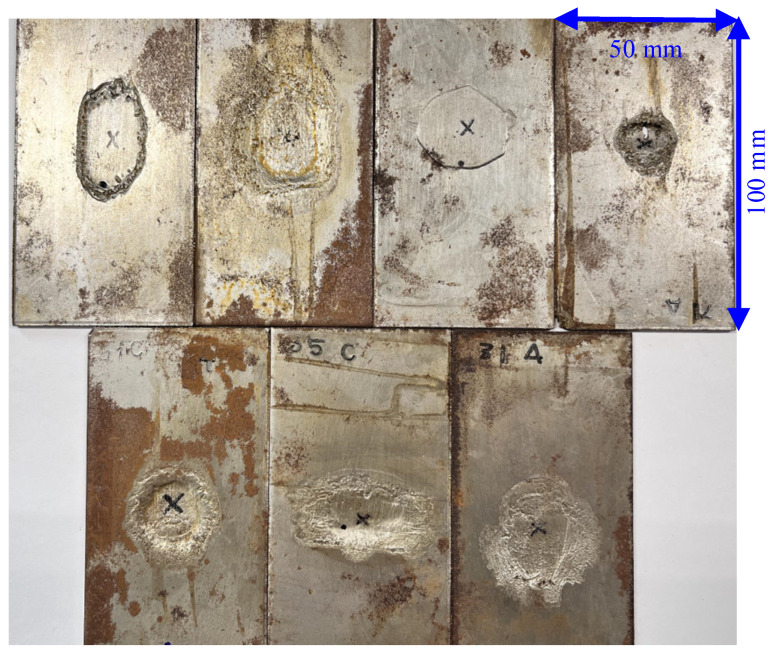
Samples with highly corroded state (state-2).

In this work, 49 SPCC steels with dimensions 100 × 50 × 2 mm^3^ are used as the samples. The samples were artificially corroded with a solution of ferric chloride to obtain different corroded states. Vinyl tape was attached to cover parts of the plate surfaces to avoid corrosion in these regions. Then, the plates were soaked into iron(iii)-chloride-based etchant H-1000a (Sunhayato Corp., Tokyo, Japan) at 50 °C to introduce corrosions [24]. The exposure time to the ferric chloride solution and the maximum depth of the corroded area for the 49 samples are shown in Figure 1. The samples are labeled as state-1 (less corroded state) when the corroded depths are smaller than 0.5 mm (25% thickness loss) which mostly corresponds to an exposure time of less than 48 h during the artificial corrosion procedure. The other samples are labeled as state-2 (highly corroded state). Figure 2 depicts a representative of seven samples belonging to the less corroded state (state-1), and a representative of seven samples belonging to the highly corroded state (state-2) is shown in Figure 3.

### 2.2. ECT Principle

When an alternating current is fed into a coil, eddy currents are generated in the conductive sample beneath it. Disturbances in the eddy currents are induced when there are defects in the material or changes in the material properties. It further leads to the changes in the magnetic field, which is measured by magnetic field sensors. The excitation coil, used to generate eddy currents in the plate, need not be in contact with the sample under test. This is especially important because prior surface preparation is not necessary.

The eddy current density $J\left(z\right)$ decays exponentially along the thickness of the plate (*z*) as:

(1) $$J\left(z\right)=J_{0}e^{-\frac{z}{\delta }},$$

where $J_{0}$ is the spatially uniform surface current density, and δ is the standard depth of penetration of eddy currents, which is:

(2) $$\delta =\sqrt{2/(\omega \mu \sigma )},$$

where ω,μ,σ are angular frequency, magnetic permeability, and electrical conductivity, respectively [25,26]. The diffusion of the eddy currents into the material thickness is accompanied by a phase delay that can be used to detect not only the existence of defects, but also their depth. Theoretically, the excitation frequency should be selected according to the depth of penetration. However, this is only true when the eddy current density is spatially uniform at the surface of a semi-infinite plate. When the plate is small and the coil is cylindrical, the equation is not accurate. Based on the previous investigations [25,26], we performed experiments at different frequencies and chose the frequencies that gave the best results.

### 2.3. Experiments

**Figure 4:**
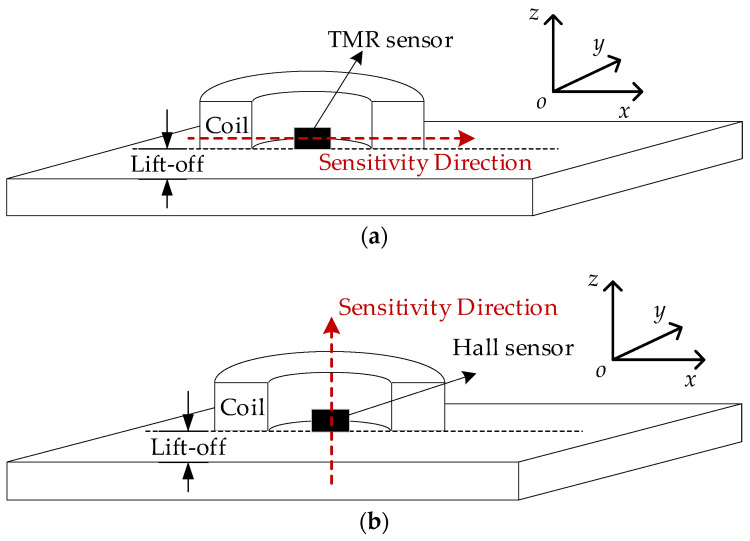
ECT probe: (**a**) Probe schematic with a TMR sensor; (**b**) Probe schematic with a Hall sensor.

**Figure 5:**
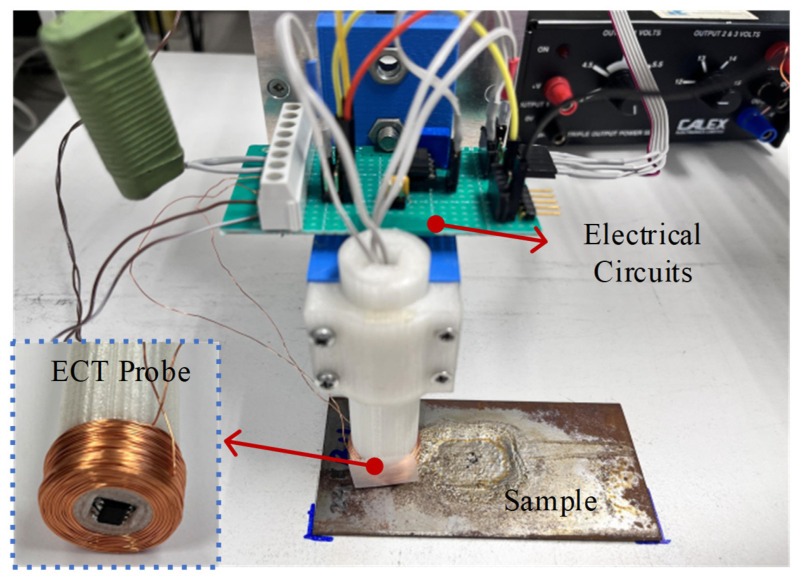
ECT probe and signal conditioning circuits.

The eddy current probe consists of an excitation coil and a magnetic field sensor, as shown in Figure 4a,b. Because the *x*-axis and *z*-axis components of the magnetic field have different magnitudes, two types of magnetic sensors are used, namely, a Hall sensor and a Tunneling Magneto Resistance (TMR) sensor. The probe with the Hall sensor is used to measure the *z*-axis component of the magnetic field *B_z_*, and the probe with the TMR sensor is used to measure *B_x_*. The fabricated ECT probe is shown in Figure 5. The excitation coil has 460 turns, and with internal radius *r* = 6 mm, external radius *R* = 12 mm, and height *H* = 10 mm. As shown in Figure 5, the plate is placed with the defects facing the ECT probe. Thus, all defects are considered to be near-side surface defects.

**Figure 6:**
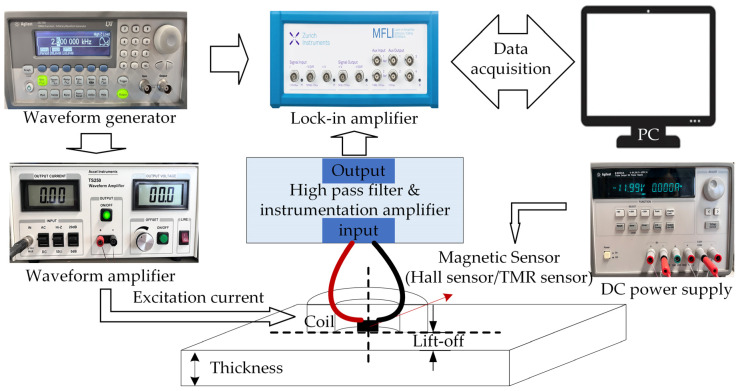
Experimental setup.

The experimental setup is illustrated in Figure 6, with the probe lift-off settled at 1 mm. A sinusoidal voltage generated by the Agilent-33210A (from Agilent, Santa Clara, CA, USA) waveform generator is injected into the excitation coil after passing through the TS250 power amplifier.

The output voltage of the sensor is processed by the signal conditioning circuit, which contains two parts: the first part is composed of a high-pass filter and an instrumentation amplifier to remove the DC bias and amplify the signal; the second part is a digital lock-in amplifier from Zurich Instruments MFLI (Zurich, Switzerland). The signal is finally displayed on the computer after the data acquisition using the acquisition board integrated within the lock-in amplifier.

In order to obtain more possible features for the machine learning models, two frequencies were used to distinguish between the smaller depth of corrosion (state-1) and the larger depth of corrosion (state-2). Smaller frequencies can penetrate deeply and hence were useful in obtaining the features from deeper pits, while larger frequencies were used for smaller surface pits. Thus, 2.5 kHz and 5 kHz sinusoidal signals were used to excite the coils based on the description in Section 2.2.

**Figure 7:**
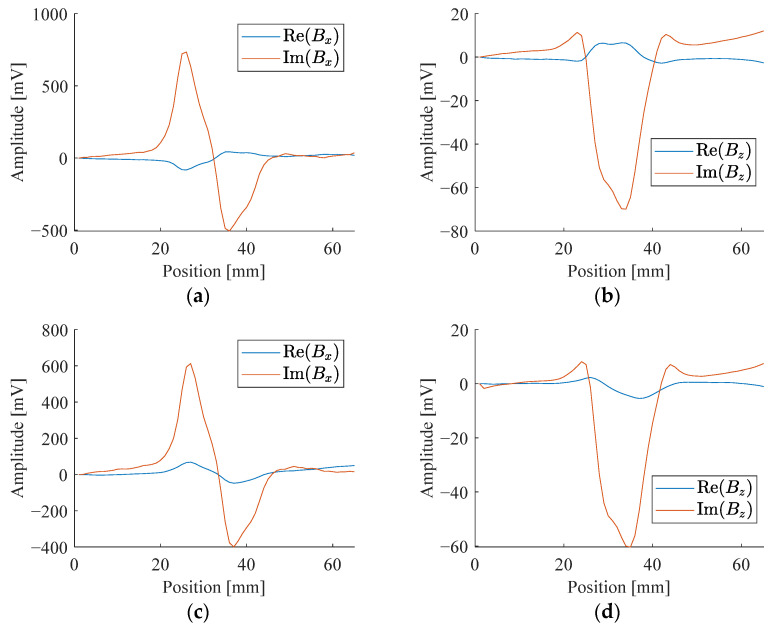
Signal response of a sample with less corroded state: (**a**) Real and imaginary parts of *B_x_* at 2.5 kHz; (**b**) Real and imaginary parts of *B_z_* at 2.5 kHz; (**c**) Real and imaginary parts of *B_x_* at 5 kHz; (**d**) Real and imaginary parts of *B_z_* at 5 kHz.

**Figure 8:**
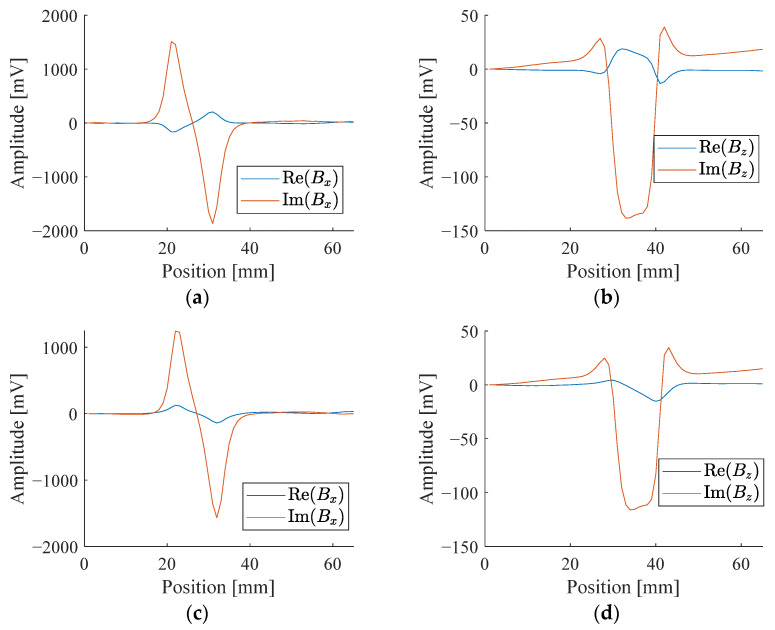
Signal response of a sample with highly corroded state: (**a**) Real and imaginary parts of *B_x_* at 2.5 kHz; (**b**) Real and imaginary parts of *B_z_* at 2.5 kHz; (**c**) Real and imaginary parts of *B_x_* at 5 kHz; (**d**) Real and imaginary parts of *B_z_* at 5 kHz.

There are three measurable components of a magnetic field. However, due to symmetry, only one horizontal component is enough to provide the information. Thus, only *B_z_* and *B_x_* are measured. For sinusoidal signals, either the amplitude-phase pair or real-imaginary pairs can be used to represent the signals. The results expressed in the two ways are interchangeable. Without loss of generality, we choose to represent the signals by real and imaginary components in this study. The real and imaginary parts of the signals are obtained from the lock-in amplifier. Figure 7 and Figure 8 show the different characteristics of *B_z_* and *B_x_* from a sample with different corroded states at these two frequencies.

The peaks of signals are chosen as the features. Since two frequencies are used for excitation, two magnetic field components are measured, and each of them has real and imaginary components; eight features can be obtained for each sample, as shown in Table 1. These features are used to train and test the generative and discriminative models. To have statistical significance in the data for the classification models, the interclass averaging technique is used to expand the dataset with 49 observations into 200 observations, with 100 observations in each class.

## 3. Classification Algorithms

Supervised machine learning algorithms can classify unknown data (test data) based on previously defined data (training data) [27,28]. There are two major perspectives in supervised machine learning techniques: a generative model (GM) and a discriminative model (DM) [29]. In its broadest sense, a GM classifier models the distribution of features in individual classes together with the class prior, and then uses Bayes’ theorem to compute the posterior distribution. Whereas a DM classifier models the posteriors directly from the learning examples. In this work, two different techniques, i.e., Gaussian mixture model (a type of GM) and logistic regression model (a type of DM), are used to classify the corrosive state of steel samples. To compare the models’ performances in identifying the corrosive states, the confusion matrix and other evaluation metrics such as accuracy, precision, recall, and F1 scores have been computed for both models.

### 3.1. Gaussian Mixture Model

**Figure 9:**
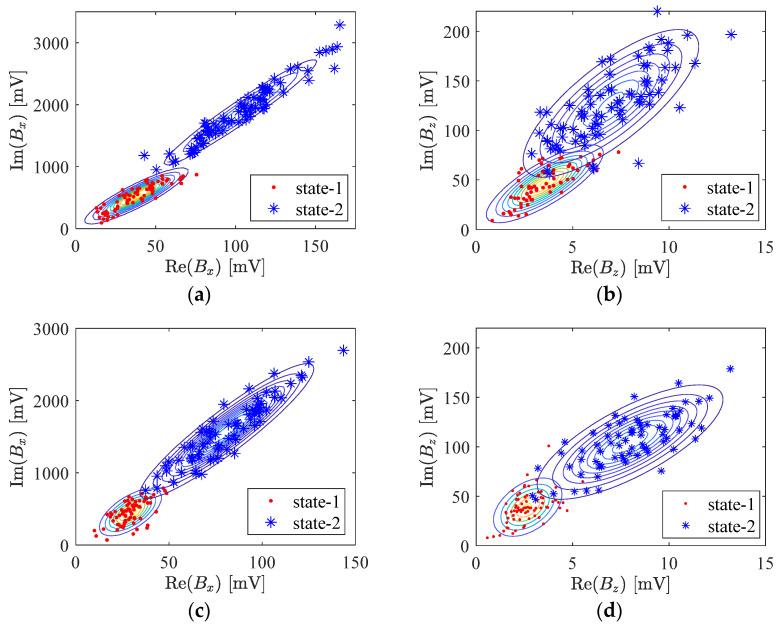
GMM model: Maximum perturbation features of (**a**) Real and imaginary parts of *B_x_* at 2.5 kHz; (**b**) Real and imaginary parts of *B_z_* at 2.5 kHz; (**c**) Real and imaginary parts of *B_x_* at 5 kHz; (**d**) Real and imaginary parts of *B_z_* at 5 kHz.

In the Gaussian mixture models (GMM), the observation sets are needed to compute the empirical mean, ${\hat{\mu }}_{j}$, and empirical covariance, $\hat{K_{j}}$, of a given class, *j*. From the mean and the covariance matrix, the probability density function for each class can be constructed as:

(3) $$x|C_{j}\sim N(\cdot ;{\hat{\mu }}_{j},\hat{K_{j}}),$$

where x represents the feature domain, $C_{j}$ represents the $j^{th}$ class. In this study, j=0 represents the state-1 of the corrosions and j=1 is the state-2. The ${\hat{\mu }}_{j}$ ($=E(x_{j})$) and $\hat{K_{j}}(=E(x_{j}^{T}x_{j}))$ are the parameters of the Gaussian distribution N. The circumflex hat indicates that the parameters are estimated empirically. For brevity and clarity, the examples of 2D data (considering only two features) of each state are shown in Figure 9.

To validate the model, out of the 100 observations of each class, 80% of them were randomly sampled (without repetition) to estimate the Gaussian parameters for each class. In this case, we have assumed the priors (the probability of occurrence of a class $C_{j}$) to be the same for each class as there is no information about the occurrence of the class. The remaining 20% of the data for each class was used to test the model. The test data were assigned to one of the classes based on the Mahalanobis distance between the test point and the Gaussian’s centers. The Mahalanobis norm for a test point $x_{t}$ is given by:

(4) $$d_{M}^{\left(j\right)}=\sqrt{{\left(x_{t}-{\hat{\mu }}_{j}\right)}^{T}{\hat{K_{j}}}^{-1}\left(x_{t}-{\hat{\mu }}_{j}\right)},$$

which is the normed distance between the test point $x_{t}$ and the center of the Gaussian distribution $N(\cdot ;{\hat{\mu }}_{j},\hat{K_{j}})$ of the *j^th^* class. Assignments to the classes are made by the following rule:

(5) $$y_{pred}^{GM}=\left\{\begin{array}{cc} 0 & \leftarrow \\ 1 & \leftarrow \end{array}\right.\begin{array}{c} d_{M}^{\left(0\right)}<d_{M}^{\left(1\right)}\\ d_{M}^{\left(0\right)}\geq d_{M}^{\left(1\right)} \end{array}.$$

### 3.2. Logistic Regression Model

For the logistic regression model, a binary classifier is represented as $f:R^{d}\to \{0,1\}$. Let the domain of the observed samples be $D=\{(x_{1},y_{1}),(x_{2},y_{2}),\ldots (x_{n},y_{n})\}$, where n is the total number of observed samples (training set). It follows Bernoulli’s distribution. The samples are independent and identically distributed with $y_{i}\in Y_{i}$, where $Y_{i}\;\sim \;f_{\left(Y|X\right)}\;(y|x_{i})$. The logistic function squashes the outputs between 0 and 1, which is represented as:

(6) $$f\left(x\right)=\frac{1}{1+e^{-w^{T}x}},$$

where the parameter *w* is the weight function that determines the slope and the shift of the logistic function. Thus, for a binary class model, the posterior distribution is written as:

(7) $$f_{Y|X}\left(y|x\right)=\left\{\begin{array}{c} \;\frac{1}{1\;+\;e^{-w^{T}x}\;}\;\leftarrow C_{1}\\ 1-f_{Y|X}\left(y=1|x\right)=\frac{1}{1\;+\;e^{w^{T}x}\;}\;\leftarrow \;C_{0} \end{array}\right..$$

In this case, $C_{0}$ corresponds to corrosion state-1 and $C_{1}$ corresponds to corrosion state-2. To estimate the *w*, it is necessary to form the likelihood function from the set of *n* independent observations. The likelihood function L(w) for the Bernoulli’s distribution is given by:

(8) $$L\left(w\right)=\prod_{i}{\left(f_{Y|X}\left(y=1|x\right)\right)}^{y_{i}}*{\left(f_{Y|X}\left(y=0|x\right)\right)}^{1-y_{i}}.$$

**Figure 10:**
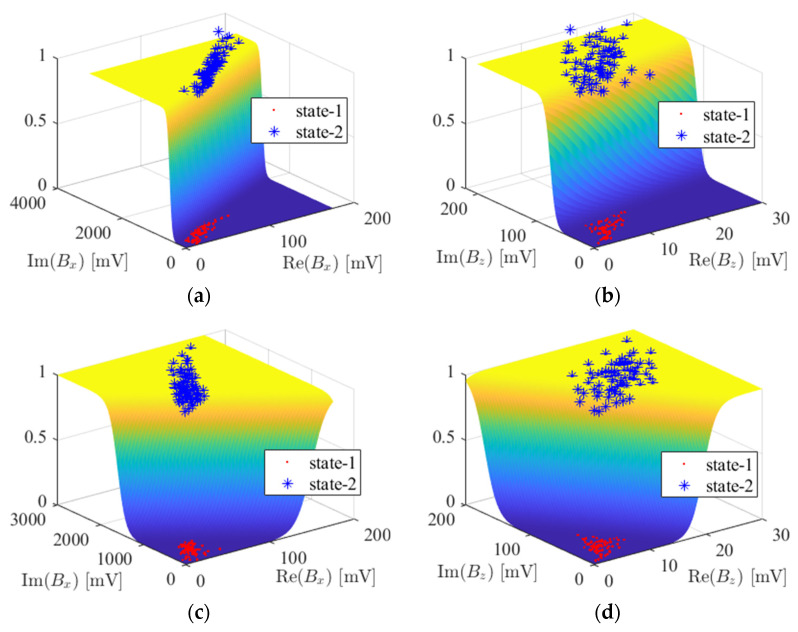
Logistic regression model: Maximum perturbation features of (**a**) Real and imaginary parts of *B_x_* at 2.5 kHz; (**b**) Real and imaginary parts of *B_z_* at 2.5 kHz; (**c**) Real and imaginary parts of *B_x_* at 5 kHz; (**d**) Real and imaginary parts of *B_z_* at 5 kHz.

As the closed-form solution for the weight estimate $\hat{w}$ cannot be found directly, generally gradient-descent algorithms are performed to obtain its optimal estimate. The main problem with this technique is the long consumption time for multiple evaluations. So, alternatively, in this work, we used the logistic regression model, expressed in log scale as $w^{T}x=log\left(y/(1-y)\right)$. The advantage of this form is the existence of a closed-form solution. However, in this case, the right-hand side is ±∞ as y is either 1 or 0. To overcome this issue, we took the class as either ϵ ($C_{0}$) or 1−ϵ ($C_{1}$), where ϵ→0. Thus, the least-squares technique could be successfully applied to obtain w. For brevity and clarity, the 2D data (considering only two features) of each state of corrosion are shown in Figure 10.

Similar to the procedure used for GMM, the observations were randomly divided into a training dataset (80% data) and a testing dataset (20% data) to validate the logistic regression model. Once the weights are found (training step), the test data $x_{t}$ are substituted into Equation (6) yielding:

(9) $$y_{pred}^{DM}=\left\{\begin{array}{cc} 0 & \leftarrow \\ 1 & \leftarrow \end{array}\right.\begin{array}{c} f\left(x_{t}\right)<0.5\\ f\left(x_{t}\right)\geq 0.5 \end{array}.$$

### 3.3. Classification Results and Discussion

**Figure 11:**
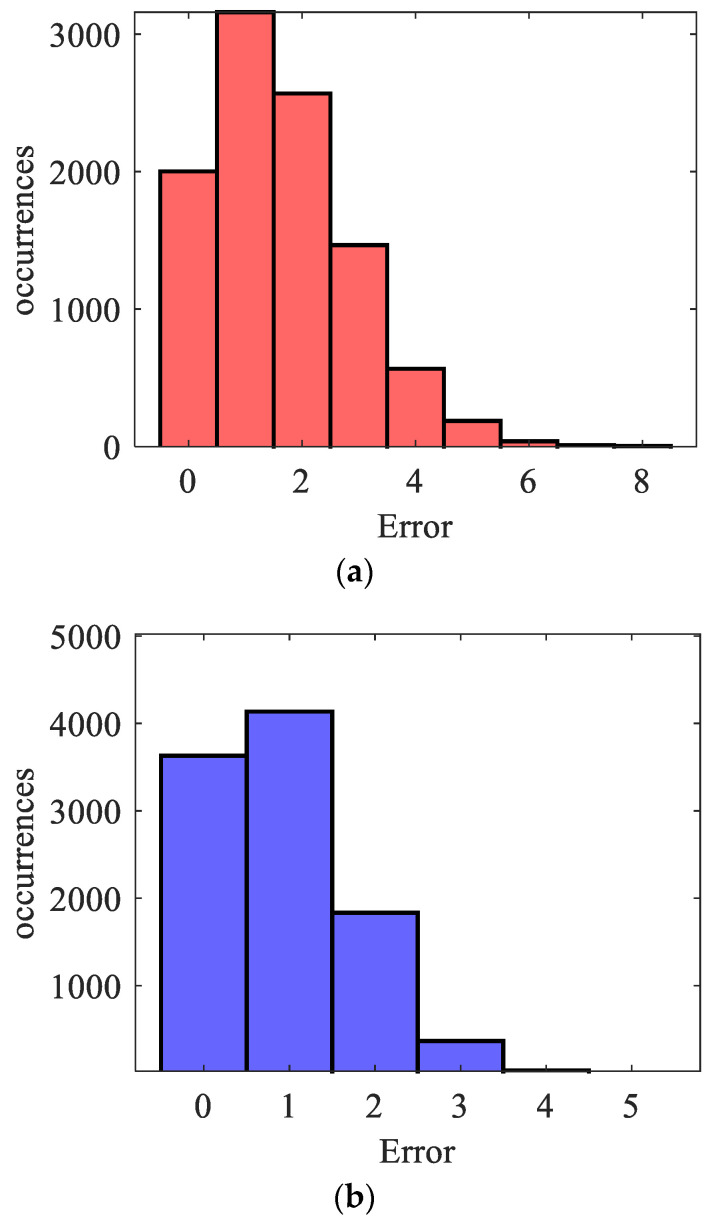
Classification errors of (**a**) GMM; (**b**) Logistic regression model.

Both the GMM and logistic regression model were evaluated for 10,000 times, where in each iteration the train data (80%) and test data (20%) for each class were randomly chosen. Once the test points were introduced, the absolute errors, $\left|y-y_{pred}^{GM/DM}\right|$, were recorded and summed to find the total errors in each iteration of the GMM and the logistic regression model. The total absolute errors with their frequencies are shown in Figure 11.

**Figure 12:**
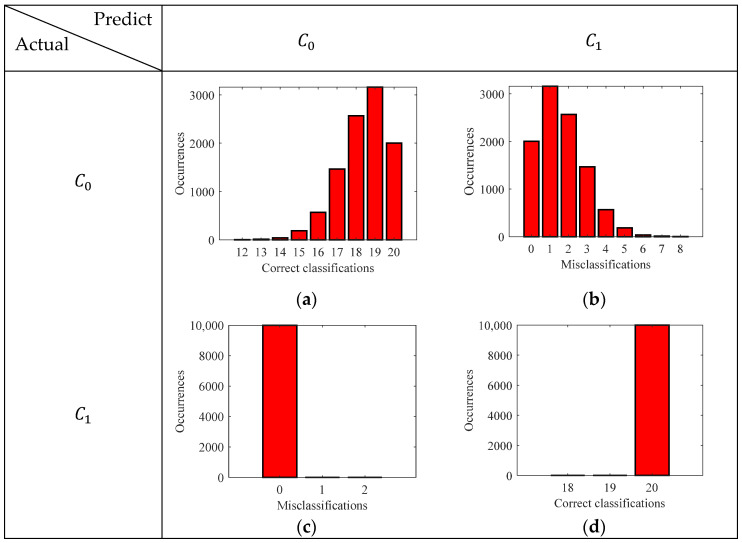
Confusion matrix depicting the correct classification and misclassification histogram GMM: (**a**) True negative; (**b**) False positive; (**c**) False negative; (**d**) True positive.

**Figure 13:**
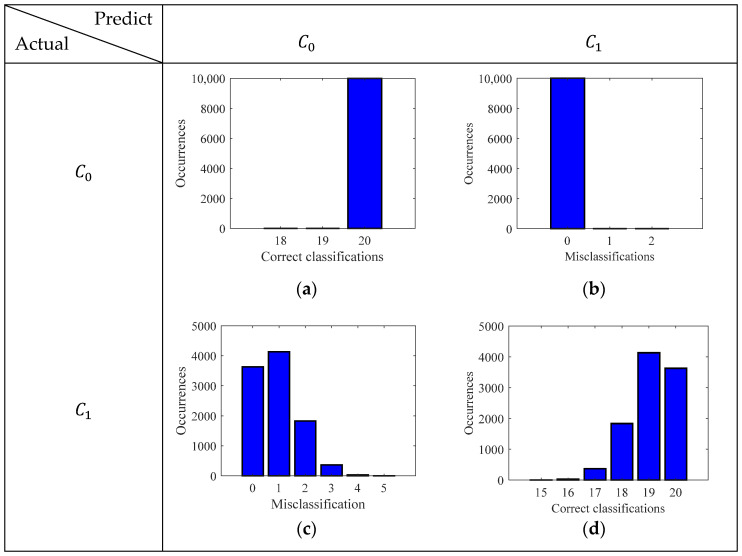
Confusion matrix depicting the correct classification and misclassification histogram logistic regression model: (**a**) True negative; (**b**) False positive; (**c**) False negative; (**d**) True positive.

It can be seen that, for the case of GMM, there was an expected error of 1.637 miscalculations in a test sample set of 40 (20 for each class). On the other hand, the logistic regression gave an expected error of 0.903 in a test sample set of 40. The absolute errors show the misclassifications of the models; however, they do not provide the details of those classes that were misclassified. Hence, to obtain the class-specific performance of the models, the confusion matrix was computed for the GMM model and logistic regression model as shown in Figure 12 and Figure 13, respectively.

**Figure 14:**
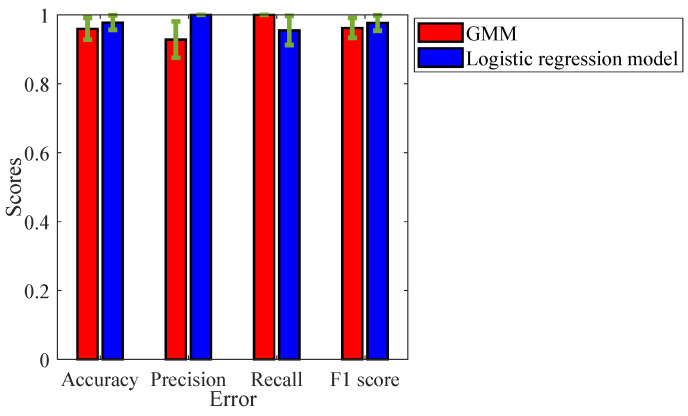
Class-specific performance metrics for the GMM and logistic regression model.

It is important to note that, for the GMM, the false negatives and true positives are 0% and 100%, respectively. Namely, the model always correctly predicts the highly corroded samples and never misclassifies them as less corroded. On the other hand, for the logistic regression model, the true negatives and false positives are always 100% and 0%, meaning that the model always correctly predicts less corroded samples. To extract further information about the class-specific performance of the models, the accuracy, precision, recall, and F1 score were derived from the confusion matrix. Figure 14 depicts these metrics for both the GMM and logistic regression model.

The accuracy of the model dictates the rate of correct classification, and the GMM can correctly classify the corrosive state of the samples 95.9% of the time with an uncertainty of 3.18%. Whereas, for the logistic regression model, the accuracy is 97.69% with an uncertainty of 2.14%. It can be seen that the recall score is 100% for the GMM model. Whereas, the recall score of the logistic regression model is not as good as the GMM. Giving a misclassification rate of 4.62% with an uncertainty of 4.2%. The recall score dictates the rate of misclassification of highly corroded samples (state-2). For the task of corrosion monitoring, it is important to have a high recall score, so that potential failures of the structure can be avoided.

Precision score dictates the rate of misclassification error of the less corroded state-1 samples (false positives). It can be seen that the precision score of the model is 100%, which means there is never a misclassification of less corroded samples. However, the GMM model does not have a satisfactory performance giving a misclassification rate of 4.62% with an uncertainty of 4.2%. The F1 score, which is basically the geometric mean between the precision and recall, is 97.63% for the GMM and 96.19% for the logistic regression model.

It can be noted that the GMM has provided a very good performance in terms of misclassification rates, hence it is potentially a good classifier to identify the highly corroded samples. On the other hand, the logistic regression model has good performance in terms of accuracy and precision. The misclassification errors due to the less corroded samples is 0, and hence it is a potentially good classifier to identify the less corroded samples.

## 4. Conclusions

Classification of corrosion states of a material is crucial because it enables the assessment of damage severity and informs tailored maintenance strategies. Hence, in this study, we have investigated the classification of the corrosive states of steel samples by supervised machine learning algorithms using features from eddy current testing. Two methodologies based on generative modeling (Gaussian mixture model) and discriminative modeling (logistic regression model) were used to classify the corrosions into two states, i.e., a less corroded state (state-1) and a highly corroded state (state-2). The eight features that were used for the classification were the peaks of the perturbations in the magnetic field components, real and imaginary parts of *B_x_* and *B_z_*, at two different frequencies. Both the models were trained with 80% of randomly selected data and tested with the remaining 20%. Further statistics on the model performance and class-specified misclassification rates, such as accuracy, precision, recall, and F1 scores, were obtained from the confusion matrix. The results lead to the following conclusions:

- Both the models, GMM and logistic regression model, can classify the corrosive state of the steel samples, using features from the perturbed magnetic flux density components.
- The GMM model has a recall score of 1, indicating that it never misclassifies the samples that are highly corroded (state-2). On the other hand, the logistic regression model occasionally misclassifies the state-2 samples.
- The logistic regression model has a precision score of 1, indicating that it never misclassifies those samples that are less corroded (state-1), while the GMM model occasionally misclassifies them.
- Both the models have a good F1 score indicating the potential application of these models to classify the corrosive state of steels.

## Figures and Tables

**Table 1:** Features used for classification.

No.	Features
1	Max(abs(Re (*B_x_*))) at 2.5 kHz
2	Max(abs(Im(*B_x_*))) at 2.5 kHz
3	Max(abs(Re (*B_z_*))) at 2.5 kHz
4	Max(abs(Im (*B_z_*))) at 2.5 kHz
5	Max(abs(Re (*B_x_*))) at 5.0 kHz
6	Max(abs(Im(*B_x_*))) at 5.0 kHz
7	Max(abs(Re (*B_z_*))) at 5.0 kHz
8	Max(abs(Im (*B_z_*))) at 5.0 kHz

## Data Availability

The data presented in this study are available on request from the corresponding author.
